# 吉非替尼对比培美曲塞二线治疗晚期非鳞型非小细胞肺癌的随机对照临床研究

**DOI:** 10.3779/j.issn.1009-3419.2013.08.03

**Published:** 2013-08-20

**Authors:** 宏宇 戴, 玲 徐, 春伟 夏, 文萍 陈

**Affiliations:** 210029 南京，南京市胸科医院呼吸科 Department of Respiratory Medicine, Nanjing Chest Hospital, Nanjing 210029, China

**Keywords:** 吉非替尼, 培美曲塞, 肺肿瘤, Geftinib, Pemetrexed, Lung neoplasms

## Abstract

**背景与目的:**

吉非替尼和培美曲塞均是晚期非小细胞肺癌（non-small cell lung cancer, NSCLC）二线治疗的药物，但直接对比两者二线治疗的研究数据有限。本研究旨在比较吉非替尼和培美曲塞二线治疗晚期非鳞型NSCLC的疗效、安全性及对生活质量的影响。

**方法:**

将46例一线含铂双药化疗方案（不含培美曲塞）治疗失败的晚期非鳞型NSCLC患者随机分为两组，每组23例，分别给予吉非替尼口服（吉非替尼组），或静脉滴注培美曲塞（培美曲塞组），比较两组的疗效和安全性及治疗对生活质量的影响。

**结果:**

培美曲塞组的客观缓解率（objective response rate, ORR）为13.0%（3/23），疾病控制率（disease control rate, DCR）为30.4%（7/23），中位无进展生存时间（median progression-free survival, mPFS）为3.1个月；吉非替尼组的ORR 17.3%（4/23），DCR 39.1%（9/23），mPFS 4.4个月；两组的ORR、DCR和mPFS均未见统计学差异（*P* > 0.05）。培美曲塞最常见的不良反应为中性粒细胞减少（n=9, 39.13%）和乏力（*n*=8, 34.78%）；吉非替尼最常见的不良反应为皮疹（*n*=8, 34.78%）和腹泻（*n*=4, 17.39%）。和治疗前基线相比，培美曲塞组和吉非替尼组治疗后生活质量评分均有不同程度的改善，吉非替尼组在情绪，活动能力及肺癌附加关注的其它因素方面较培美曲塞组改善更明显（*P* < 0.05）。

**结论:**

吉非替尼和培美曲塞二线治疗晚期非鳞型NSCLC的疗效相似，不良反应各异；两者均能改善患者的生活质量，但是吉非替尼改善更明显。

## Abstract (en)

**Background and objective:**

Geftinib and Pemetrexed are drugs used as second-line therapy for advanced non-small cell lung cancer (NSCLC), although studies comparing the two drugs are limited. The aim of this study is to explore the effects, safety, and quality of life (QoL) of Geftinib and Pemetrexed on patients with advanced non-squamous NSCLC.

**Methods:**

Forty-six advanced non-squamous NSCLC patients who failed to frst-line therapy were randomly divided into two groups with 23 patients each, one using oral Geftinib (Geftinib group) and the other using intravenous injection Pemetrexed (Pemetrexed group). The effects, safety, and QoL were determined and analyzed.

**Results:**

For the Pemetrexed group, objective response rate (ORR) was 13.0% (3/23), disease control rate (DCR) was 30.4% (7/23), and median progression-free survival (mPFS) was 3.1 months. In the Geftinib group, ORR was 17.3% (4/23), DCR was 39.1% (9/23), and mPFS was 4.4 months. Compared with the Pemetrexed group, the ORR, DCR, and mPFS in the Geftinib group exhibited no statistical signifcance (*P* > 0.05). Furthermore, the most common toxicities in the Pemetrexed group were neutropenia (*n*=9, 39.13%) and fatigue (*n*=8, 34.78%), whereas those in the in Geftinib group were skin rash (*n*=8, 34.78%) and diarrhea (*n*=4, 17.39%). Compared with baseline, the QoL improved in both groups but to different degrees. Likewise, emotional, functional wellbeing, and QoL aspects specifcally related to lung cancer were beter improved in the Geftinib group than in the Pemetrexed group (*P* < 0.05).

**Conclusion:**

The effects of Pemetrexed and Geftinib as second line therapy were similar, although with different AEs. Both drugs could improve the QoL, but Geftinib showed beter overall results than Pemetrexed.

肺癌是发病率和死亡率均居首位的恶性肿瘤，并且发病率呈上升趋势^[1]^。在肺癌中，大约75%的患者为非小细胞肺癌(non-small cell lung cancer, NSCLC)，而50%左右的NSCLC在初始诊断时即为晚期或已转移^[2]^。2004年WHO的肺癌分类将NSCLC分为鳞癌、腺癌、大细胞癌、腺鳞癌和类癌等。最新版NCCN指南推荐对晚期NSCLC先需要明确病理组织类型，针对不同病理类型进行后续的规范检测和治疗选择。多西他赛或培美曲塞单药化疗，或小分子酪氨酸激酶受体抑制剂(epidermal growth factor receptor tyrosine kinase inhibitor, EGFR-TKI)(吉非替尼或厄洛替尼)是推荐的非鳞型NSCLC的二线治疗方案^[3]^。已有两项大型Ⅲ期临床研究分别证实，培美曲塞和吉非替尼二线治疗的疗效和多西他赛相似，安全性更好^[4, 5]^，但是直接对比吉非替尼和培美曲塞二线治疗晚期NSCLC的研究数据有限^[6]^。本研究旨在前瞻性观察吉非替尼和培美曲塞二线治疗晚期非鳞型NSCLC的疗效和安全性，并比较两者对患者生活质量的影响，从而为临床二线药物的合理选择提供参考依据。

## 1 材料与方法

### 1.1 研究对象

选择2010年1月-2012年8月在南京胸科医院接受治疗的符合入组标准的晚期NSCLC患者46例，随机分为两组，培美曲塞组和吉非替尼组。入组标准如下：①病理组织学或细胞学确诊的非鳞型NSCLC；②临床分期为Ⅲb期或Ⅳ期；③均经含铂方案(不含培美曲塞)化疗4个-6个周期后失败；④至少具有一个或以上的可评价靶病灶；⑤ECOG PS评分0分-2分；⑥年龄18岁-75岁；⑦足够的骨髓和器官功能。所有患者入组前均签署知情同意书。

### 1.2 治疗及评价方法

培美曲塞组：500 mg/m^2^，静脉滴注，第1天使用，每21天为一个周期重复，直至疾病进展或出现不可耐受的毒副作用。在应用培美曲塞24 h前口服地塞米松，每天两次，每次4 mg，连服3天；首次用培美曲塞前一周给予维生素B12 1, 000 μg肌肉注射，每3个周期重复一次；首次用培美曲塞一周前口服金施尔康(每片含叶酸400 μg)，每天一片，直至培美曲塞结束后21天。吉非替尼组：250 mg，口服，一日一次，连续服用至疾病进展或出现不可耐受的毒副反应。两组均每6周进行影像学评价疗效。参照RECIST 1.1实体瘤疗效评价标准进行近期疗效评价，分为完全缓解(complete response, CR)、部分缓解(partial response, PR)、疾病稳定(stable disease, SD)和疾病进展(progressive disease, PD)，以CR+PR为客观缓解率(objective response rate, ORR)。无进展生存时间(progression-free survival, PFS)为自随机化分组开始至肿瘤进展或死亡的时间。治疗毒性反应评价参照CTCAE 3.0标准分为0度-Ⅳ度共5个级别。采用FACT-L量表进行生活质量的评估，包含日常活动(7项)、社会/家庭生活(7项)、情绪(6项)、活动能力(7项)和肺癌附加关注的其它因素(9项) 5个方面。每项均采用五级评分法：一点也不；有一点点；有几分；颇有一些；非常多。评分时正向项直接计0分-4分，逆向项则反向计分，将各项得分相加即为总分。分别在治疗前进行基线评价，之后每个治疗周期后进行评价，直至疾病进展或连续评价6个周期。

### 1.3 统计学处理

采用SPSS 13.0统计分析软件分析数据，对计量资料进行*t*检验或秩和检验，计数资料进行*χ*^2^检验，以*P* < 0.05为差异有统计学意义。

## 2 结果

### 2.1 患者的基线特征

两组患者的基本临床特征见表 1，两组患者在性别、年龄、PS评分、吸烟状态、病理亚型及疾病分期均未见统计学差异(*P* > 0.05)，两组患者的基线特征平衡，具有可比性。

**1:** 两组患者的基本临床特征 The basic clinical characteristics of patients in the two groups

Characteristics	Pemetrexed (*n*=23)	Gefitinib (*n*=23)
Gender		
Male/Female	14/9	15/8
Age, yr, median (range)	61 (47-72)	62 (41-74)
Performance status		
0/1/2	6/12/5	6/11/6
Smoking status		
Current or ever-smoker/Never-smoker	12/11	13/10
Pathologic subtype		
Adenocarcinoma	21	22
Large-cell carcinoma	1	1
Other	1	0
Initial stage		
Ⅲb/Ⅳ	5/18	4/19

### 2.2 疗效评价

两组患者的治疗疗效评价见表 2。培美曲塞组：ORR 13.0%(3/23)，疾病控制率(disease control rate, DCR) 30.4% (7/23)，mPFS为3.1个月；吉非替尼组：ORR 17.3%(4/23)，DCR 39.1%(9/23)，mPFS为4.4个月。两组的ORR、DCR和mPFS均未见统计学差异(*P* > 0.05)，无进展生存时间的*Kaplan-Meier*生存曲线见图 1。

**2:** 两组患者治疗疗效的比较 Comparison of the efficacy of treatments in the two groups

Group	*n*	CR (%)	PR (%)	SD (%)	PD (%)	ORR (%)	DCR (%)	mPFS (month)
Pemetrexed	23	0(0)	3(13.0)	4(17.3)	16 (69.6)	3 (13.0)	7 (30.4)	3.1
Gefitinib	23	1 (4.3)	3 (13.0)	5(21.7)	14 (60.9)	4(17.3)	9 (39.1)	4.4
Statistic						*u*=0.4060	*u*=0.6199	*t*=1.4728
*P*						> 0.05	> 0.05	> 0.05
CR: complete response; PR: partial response; SD: stable disease; PD: progressive disease; ORR: objective response rate; mPFS: median progression-free survival.								

**1:**
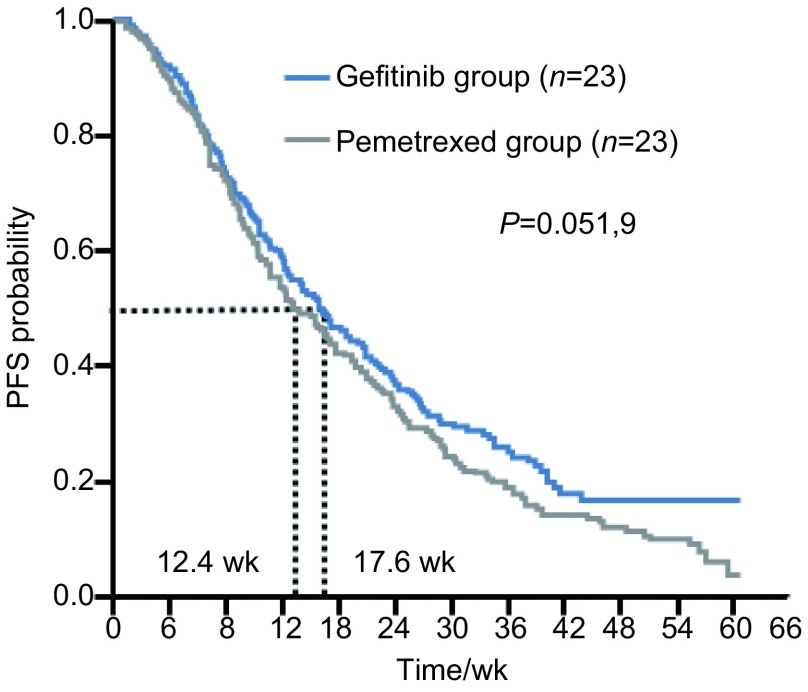
无进展生存时间的*Kaplan-Meier*生存曲线 *Kaplan-Meier* survival curve of progressive-free survival (PFS)

### 2.3 不良反应评价

46例患者均可进行毒性评估，在培美曲塞组，中位治疗周期为2(范围1-10)，中位治疗时间为2.9个月，在吉非替尼组，中位治疗时间是3.7个月，两组的中位治疗时间未见统计学差异(*P* > 0.05)。在培美曲塞组，最常见的不良反应为中性粒细胞减少(*n*=9, 39.13%)和乏力(*n*=8, 34.78%)；在吉非替尼，常见的不良反应为皮疹(*n*=8, 34.78%)和腹泻(*n*=4, 17.39%)，见表 3。这些不良反应多为轻中度，经临床对症支持治疗均能得到有效的控制。

**3:** 两组患者治疗不良反应的比较 Comparison of the toxicity of treatments in the two groups

Adverse event (G1-4/G3-4)	Pemetrexed (*n*=23)	Gefitinib (*n*=23)
Hematologic toxicity		
Anemia	4/0	1/0
Neutropenia	9^Δ^/1	2/0
Thrombocytopenia	2/0	0/0
Non-hematologic toxicity		
Fatigue	8^□^/1	1/0
Anorexia	5/1	2/0
Nausea/vomiting	3/0	2/0
Constipation	2/0	0/0
Diarrhea	0/0	4^☆^/1
Stomatitis	1/0	1/1
Skin disorders	1/0	8^※^/2
Elevated transaminas	4/0	2/0
Fever	3/0	0/0
Compared with Gefitinib, ^Δ^*χ*^2^=5.8545, *P* < 0.05; ^□^*χ*^2^=6.0919, *P* < 0.05; Compared with Pemetrexed, ^☆^*χ*^2^=4.3810, *P* < 0.05; ^※^*χ*^2^=6.0919, *P* < 0.05.		

### 2.4 生活质量评估

和治疗前基线相比，培美曲塞组和吉非替尼组治疗后生活质量评分均有不同程度的改善，差异均具有统计学意义(*P* < 0.05)。吉非替尼组在情绪、活动能力及肺癌附加关注的其它因素方面较培美曲塞组改善更明显，其差值比较具有统计学意义(*P* < 0.05)；两组的总评分较基线值均有改善(*P* < 0.05)，但吉非替尼组的增幅更大(*P* < 0.05)，见表 4。

**4:** 两组患者生活质量评分 Comparison of the quality of life in the two groups

Group	*n*	Time	Physical well-being	Social/family well-being	Emotional well-being	Functional well-being	Other facts (lung cancer additional)	Total
Pemetrexed	23	Before therapy	18.42±3.71	14.64±2.62	13.94±3.83	12.29±3.72	23.84±3.56	92.86±8.49
		After therapy	21.17±4.49	17.26±3.97	17.02±4.13	14.58±3.96	26.49±3.48	105.41±7.53
*t*			2.265, 5	2.641, 6	2.622, 5	2.022, 9	2.459, 3	2.241, 5
*P*			< 0.05	< 0.05	< 0.05	< 0.05	< 0.05	< 0.05
Gefitinib	23	Before therapy	20.21±4.53	15.04±5.05	15.17±4.94	13.13±4.25	23.77±7.61	96.76±11.54
		After therapy	23.16±4.91	18.42±4.73	18.42±4.28	16.85±5.79	27.83±5.42	109.14±21.92
*t*			2.117, 8	2.342, 7	2.384, 6	2.483, 9	2.084, 1	2.396, 8
*P*			< 0.05	< 0.05	< 0.05	< 0.05	< 0.05	< 0.05
Pemetrexed	23	*D*-value before therapy	5.52±3.83	5.37±3.74	4.56±3.97	5.24±3.51	5.05±5.84	19.44±2.27
Gefitinib	23	*D*-value after therapy	6.93±2.56	6.99±4.72	6.94±3.85	7.58±4.26	8.67±6.03	31.47±2.61
*t*			1.467, 9	1.290, 1	2.064, 0	2.033, 2	2.068, 1	
*P*			> 0.05	> 0.05	< 0.05	< 0.05	< 0.05	< 0.05

## 3 讨论

肺癌是全球和我国发病率和死亡率均居首位的恶性肿瘤，并且发病率呈上升趋势^[1]^。NCCN指南推荐对*EGFR*基因突变阴性和ALK阴性或状态未知的晚期NSCLC患者，含铂双药化疗或联合抗血管生成药物是一线标准治疗方案，多西他赛或培美曲塞单药化疗或EGFR-TKI (吉非替尼或厄洛替尼)是二线推荐治疗方案^[3]^。培美曲塞是一种多靶点抗代谢药物，是叶酸类似物，通过破坏细胞内叶酸依赖性的正常代谢过程，抑制细胞复制，从而抑制肿瘤的生长。研究显示，培美曲塞能够抑制胸苷酸合成酶、二氢叶酸还原酶和甘氨酰胺核苷酸甲酰转移酶的活性，这些酶都是合成叶酸所必需的酶，参与胸腺嘧啶核苷酸和嘌吟核苷酸的生物再合成过程，培美曲塞可以从多个途径抑制嘧啶和嘌呤的合成，造成叶酸代谢和核苷酸合成过程的异常，从而起到抗肿瘤的作用^[7]^。一项非劣效Ⅲ期临床研究结果显示，培美曲塞二线治疗晚期NSCLC和多西他赛相比疗效相似，安全性更好^[4]^。吉非替尼是一种口服的小分子EGFR-TKI，其作用机制主要是通过抑制EGFR自身磷酸化而阻滞传导，抑制肿瘤细胞的增殖，实现靶向治疗^[8]^。一项Ⅲ期临床研究^[5]^ (INTEREST)结果显示，对一线含铂方案治疗失败的晚期或转移性NSCLC，吉非替尼和多西他赛相比，疗效相似，服药方便，患者耐受性更好。虽然这些药物是临床二线常用的方案，但是直接对比吉非替尼和培美曲塞二线治疗晚期NSCLC的研究数据有限^[6]^。

一项前瞻性Ⅲ期临床研究^[9]^结果显示，培美曲塞对非鳞癌疗效优于鳞癌，而IPASS研究^[10]^证实，吉非替尼对不吸烟腺癌的优势人群具有更好的疗效。近年来，随着肿瘤个体化治疗的发展，已经发现*EGFR*基因突变是吉非替尼有效的疗效预测因子，而优势人群的本质是不吸烟腺癌患者*EGFR*基因突变比率较高，达到59.7%^[10]^。根据肿瘤生物标记物来选择治疗是大势所趋，然而在现有的临床工作中，二线治疗前再次获取肿瘤标本困难重重，包括患者的意愿，经济因素，取材困难以及肿瘤标本异质性等因素，因此根据临床特征来给患者选择合适的二线治疗方案意义很大。*EGFR*基因突变检测的方法主要有直接测序法和ARMS法，目前患者需要自费检测，本研究入组的患者均按照NCCN指南要求，建议进行*EGFR*基因突变检测，但由于患者意愿、经济因素等的限制，愿意进行检测的仅有个别患者，因此未对*EGFR*基因突变状态进行分析。本研究选择非鳞型晚期NSCLC作为研究对象，头对头比较培美曲塞和吉非替尼治疗的疗效和安全性，研究结果显示，培美曲塞组的ORR 13.0%(3/23)，DCR 30.4%(7/23)，mPFS为3.1个月；吉非替尼组的ORR 17.3%(4/23)，DCR 39.1%(9/23)，mPFS为4.4个月。两组的ORR、DCR和mPFS均未见统计学差异。韩国一项Ⅲ期临床研究^[11]^采用吉非替尼和培美曲塞治疗既往一线含铂方案治疗失败的不吸烟晚期肺腺癌患者，结果显示，吉非替尼的mPFS优于培美曲塞(9.0个月*vs* 3.0个月，*P*=0.000, 6)。这与本研究结果不一致，可能与该研究入组人群为不吸烟腺癌患者，而本研究入组为晚期非鳞型NSCLC有关。TKI治疗*EGFR*突变患者的有效率达到了70%以上，IPASS研究^[10]^显示，不吸烟腺癌患者*EGFR*基因突变率为59.7%，吉非替尼治疗的有效率为47%，而非鳞癌患者的*EGFR*突变比例约为30%。

培美曲塞最常见的不良反应为中性粒细胞减少(*n*=9, 39.13%)和乏力(*n*=8, 34.78%)；吉非替尼最常见的不良反应为皮疹(*n*=8, 34.78%)和腹泻(*n*=4, 17.39%)，这些不良反应多为轻中度，经临床对症支持治疗均能得到有效的控制。

在我国，80%以上的肺癌患者在诊断时已属晚期，失去手术治疗的机会，药物治疗成为主要的姑息治疗手段，以往的治疗疗效评价多采用客观有效率，生存时间等指标进行量的分析，往往忽视了患者自身的感受。近年来，随着医学模式由生物医学向生物-心理-社会医学模式的转变，生活质量在肿瘤学领域越来越受到重视，已经成为国际公认的临床治疗终点目标之一，生活质量也是对患者进行整体状况评估、预后预测及疗效评价必要的参考指标^[12, 13]^。本研究采用FACT-L量表来评估两种治疗方案对患者生活质量的影响，结果显示，和治疗前基线状态相比，培美曲塞和吉非替尼均能不同程度地改善患者的生活质量，和培美曲塞相比，吉非替尼在情绪、活动能力及肺癌附加关注的其它因素方面较培美曲塞改善更明显，提示两种药物均能改善患者的生活质量，而吉非替尼改善更明显。

综上所述，吉非替尼和培美曲塞二线治疗晚期非鳞型NSCLC的近期疗效相似(ORR、DCR、mPFS)，不良反应各异，培美曲塞最常见的不良反应为中性粒细胞减少和乏力；吉非替尼最常见的不良反应为皮疹和腹泻，并且多为轻中度，经临床对症支持治疗均能得到有效的管理。培美曲塞和吉非替尼均能改善患者的生活质量，但是吉非替尼的改善更明显。但由于本研究样本量有限，还有待进一步扩大样本量进行更全面分析，为临床治疗选择提供更多的理论依据。
